# 

**Published:** 1895-12

**Authors:** 


### TABLE OF CONTENTS.

ORIGINAL COMMUNICATIONS. PAGE

Deformities of the Hard Palate in Degenerates. By FREDERICK PETERSON, M.D. . . . . . . . . . . . . . . . . . . . . . . . . . . . . . . . 719

A New Apparatus for continuing Anæsthesia while Operating in the Mouth. By THOMAS FILLEBROWN, M.D., D.M.D., Boston, Mass. . . . 731

Assistance and Assistants. By C. A. BRACKETT, D.M.D., Newport, R. I. . 736

The Heroic in Dental Operation, as contrasted with Conservatism. By C. V. KRATZER, D.D.S., Reading, Pa. . . . . . . . . . . . . . . . . . 744

Treatment of Decay in Approximate Surfaces of Bicuspids and Molars. By JULIUS G. W. WERNER, D.M.D., Boston, Mass. . . . . . . . . . . . 747

REPORTS OF SOCIETY MEETINGS.

New York Odontological Society . . . . . . . . . . . . . . . . . . . . . 752

American Academy of Dental Science . . . . . . . . . . . . . . . . . . 763

Academy of Stomatology . . . . . . . . . . . . . . . . . . . . . . . . 770

EDITORIAL.

Spurious History . . . . . . . . . . . . . . . . . . . . . . . . . . . 777

The Close of Volume XVI. . . . . . . . . . . . . . . . . . . . . . . . 780

Golden Anniversary Celebration . . . . . . . . . . . . . . . . . . . . 782

BIBLIOGRAPHY . . . . . . . . . . . . . . . . . . . . . . . . . . . . . 782

CURRENT NEWS.

Report of Treasurer of World's Columbian Dental Congress in Full from March 23, 1891, to July 23, 1895 . . . . . . . . . . . . . . . . . . 783

Massachusetts Dental Society . . . . . . . . . . . . . . . . . . . . . 786

Golden Anniversary Celebration . . . . . . . . . . . . . . . . . . . . 786

